# Low-density Lipoprotein-Cholesterol Lowering Strategies for Prevention of Atherosclerotic Cardiovascular Disease: Focus on siRNA Treatment Targeting PCSK9 (Inclisiran)

**DOI:** 10.1007/s11886-020-01427-6

**Published:** 2020-10-21

**Authors:** David Sinning, Ulf Landmesser

**Affiliations:** 1 Department of Cardiology, Charité – University Medicine Berlin (Campus Benjamin Franklin), Berlin, Germany; 2 Berlin Institute of Health (BIH), Hindenburgdamm 30, 12203 Berlin, Germany; 3 German Centre for Cardiovascular Research (DZHK), Partner Site Berlin, Berlin, Germany

**Keywords:** siRNA, Hypercholesterolemia, LDL-C, PCSK9, Coronary disease

## Abstract

**Purpose of Review:**

The aim of low-density lipoprotein-cholesterol (LDL-C) lowering therapies is to safely achieve a consistent and long-term reduction in exposure of the vasculature to atherogenic lipoproteins in order to reduce the risk of atherosclerotic cardiovascular (CV) disease and the associated CV events, such as myocardial infarctions and ischemic strokes. This review summarizes the concept and clinical development of a novel molecular approach to efficiently lower LDL-C, a synthetic small interfering ribonucleic acid (siRNA)—inclisiran—directed against proprotein convertase subtilisin-kexin type 9 (PCSK9).

**Recent Findings:**

The understanding of genes regulating atherogenic lipoproteins and their causal role in the development of atherosclerotic CV disease has substantially advanced over the past years. This has opened the possibility for development of molecular therapies targeting these atherogenic lipoproteins, in particular by RNA-targeted treatment approaches. The most advanced clinical development program is the siRNA-treatment targeting PCSK9 (inclisiran), involving more than 4000 patients in clinical studies. Phase 1 and 2 studies have identified the dose of 300 mg inclisiran for efficient LDL-C lowering. Most recently, three phase 3 studies demonstrated that a regimen of inclisiran every 6 months was feasible and reduced LDL-C by approximately 50% in patients at high or very high CV risk or with familial hypercholesterolemia. Adverse events were similar in the inclisiran and the placebo groups, except for more frequent transient injection site reactions with inclisiran than with placebo.

**Summary:**

siRNA therapy targeting PCSK9 (inclisiran) applied twice a year efficiently reduced LDL-C by approximately 50% and was safe in recent phase 3 studies. The effects of this treatment on CV outcome are currently further assessed in a large ongoing CV outcome trial.

## Introduction

Improved preventive and therapeutic strategies for atherosclerotic cardiovascular disease (ASCVD) have led to a decrease in age-adjusted mortality from ASCVD [1, 2]. ASCVD remains, however, a leading cause of morbidity and mortality globally [3–5]. In Europe, ASCVD is considered to cause more than 4 million deaths annually and is thought to be accountable for up to 45% of all deaths [4].

Dyslipidemia represents a particular relevant risk factor for ASCVD: in the INTERHEART case-control study with 27.098 participants, an elevated apolipoprotein B/apolipoprotein A1 ratio was associated with the highest odds ratio for myocardial infarction among all modifiable CV risk factors [6]. The fact that elevated levels of low-density lipoprotein-cholesterol (LDL-C) play a causal role in the development of ASCVD is well accepted, given the results of numerous epidemiological and genetic studies, as well as randomized controlled clinical trials (RCTs) [7–10, 11•]. The positive association between LDL-C and ASCVD extends even to very low levels of LDL-C [12]. While the overall CV risk is considerably low in populations with genetically determined low levels of LDL-C [13], lifetime exposure to elevated LDL-C is associated with an excessive risk of ASCVD, e.g., in patients with familial hyperlipoproteinemia (FH) [14].

## Therapeutic Approaches in the Treatment of Hypercholesterolemia

Statins have become a primary therapeutic cornerstone in ASCVD prevention since they have been shown to reduce CV events by reducing levels of LDL-C [15]. But despite the proven efficacy and safety of statins, several aspects indicate the substantial need for additional or alternative LDL-C lowering therapies.

First of all, a considerably high variability in individual responses to statin therapy has been shown [16]. Furthermore, possible side effects of statin therapy such as statin associated muscle symptoms become relevant by potentially reducing adherence to treatment. Consequently, side effects of statin therapy represent a strong predictor of failure to meet LDL-C goals [17, 18]. Observational data suggest that as many as half of patients in whom statin therapy is initiated will discontinue it within 1 year [19]. Moreover, a substantially elevated risk for CV events remains in a large proportion of patients, especially in those with persistent elevation of LDL-C levels despite a maximum tolerated dose of statin therapy [20].

Ezetimibe lowers LDL-C by preventing cholesterol absorption through inhibition of the Niemann-Pick C1-Like 1 protein. The results of the IMPROVE-IT trial showed that, after ACS, adding ezetimibe to statin therapy to lower LDL-C below levels achieved by statin therapy alone is safe and further improved CV outcome [21].

## The Role of PCSK9 in the Treatment of Hypercholesterolemia

In 2003, Abifadel and colleagues reported a gain of function mutation of proprotein convertase subtilisin-kexin type 9 (PCSK9) causing FH [22]. Subsequently, it was reported that sequence variations in PCSK9 with lower levels of LDL-C were associated with a reduced risk of coronary disease [23–25]. PCSK9 is synthesized and secreted from the liver and binds LDL receptors both intracellularly and extracellularly, promoting their lysosomal degradation in hepatocytes and thereby increased levels of circulating LDL [26]. Consequently, PCSK9 was considered a highly promising target for LDL-C lowering and preventive therapies [27].

## PCSK9 Antibody Therapy Prevents PCSK9 from Binding to the LDL Receptor

Initially, therapeutic approaches to lower circulating levels of PCSK9 have focused on the use of monoclonal antibodies. These antibodies sequester PCSK9 in the reticuloendothelial system, preventing it to bind to the LDL receptor [28, 29]. Recently, two large RCTs have shown that subcutaneous administration of a monoclonal PCSK9 antibody safely reduced both LDL-C levels and CV events in patients at very high CV risk [30, 31]. PCSK9 antibodies need to be administered by subcutaneous injections once or twice monthly [27].

## RNA-Targeted Therapy to Prevent Hepatic Synthesis of PCSK9

**Fig. 1:**
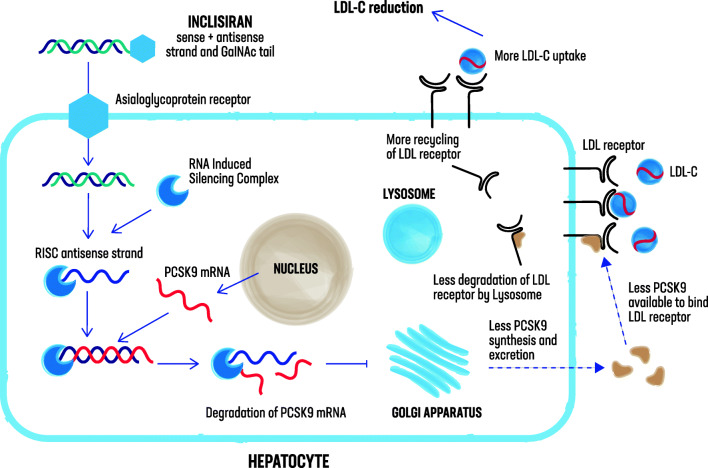
Inclisiran: mechanism of action. Inclisiran is a long-acting, synthetic siRNA directed against proprotein convertase subtilisin-kexin type 9 (PCSK9), conjugated to triantennary N-acetylgalactosamine carbohydrates, which bind to abundant liver-expressed asialoglycoprotein receptors, leading to a rapid uptake of inclisiran specifically into hepatocytes. Inclisiran engages the natural pathway of RNA interference (RNAi) by binding to the RNA-induced silencing complex (RISC) intracellularly, enabling it to catalytically cleave PCSK9 messenger RNA (mRNA), which itself is then degraded, inhibiting translation into PCSK9. With less PCSK9 available, more low-density lipoprotein-cholesterol (LDL-C) receptors can be recycled to the hepatic membrane for LDL-C uptake

siRNA molecules engage the natural pathway of RNA interference (RNAi) by binding to the RNA-induced silencing complex (RISC) intracellularly, enabling it to catalytically cleave messenger RNA (mRNA), which itself is then degraded and translation into protein is inhibited [32, 33] (Fig. 1). Previous findings have demonstrated the ability of RNAi therapeutic agents to inhibit the synthesis of liver-derived target proteins in diseases such as transthyretin amyloidosis and cancer with liver involvement [34, 35]. Since circulating PCSK9 is largely derived from the liver, targeting its hepatic production through administration of a siRNA molecule offers an alternative to the use of monoclonal antibodies.

In a first randomized, single-blind, placebo-controlled phase 1 trial with 32 healthy volunteers, the administration of ALN-PCS, an intravenous formulation of a PCSK9 synthesis inhibiting siRNA, led to both a reduction in the levels of PCSK9 and the concentration of serum LDL-C [36].

## Inclisiran Is a Subcutaneously Delivered siRNA Directed Against PCSK9

Inclisiran (ALN-PCSsc) is a long-acting, synthetic siRNA directed against PCSK9, conjugated to triantennary N-acetylgalactosamine carbohydrates, which bind to abundant liver-expressed asialoglycoprotein receptors, leading to a rapid uptake of inclisiran specifically into hepatocytes after subcutaneous injection [37] (Fig. 1). Other than ALN-PCS (intravenous formulation), inclisiran is delivered subcutaneously.

## Safety, Side Effect Profile, and Pharmacodynamic Effects of Inclisiran

Safety, side effect profile, and pharmacodynamic effects of PCSK9 siRNA were assessed in another phase 1 trial [38]. In this single-blind study, a total of 69 volunteers were randomized in a 3:1 ratio to receive either a subcutaneous injection of inclisiran or placebo in either a single ascending dose (25, 100, 300, 500, or 800 mg) or a multiple-dose phase (125 mg weekly for four doses, 250 mg every other week for two doses, or 300 or 500 mg monthly for two doses, with or without concurrent statin therapy). Participants had to have an LDL-C level of at least 100 mg/dl and a fasting triglyceride level of at least 400 mg/dl. Furthermore, participants with a history of CV disease, cerebrovascular disease, or diabetes were excluded, except for those on a stable statin therapy. Such patients could be enrolled if they had non-insulin-dependent diabetes mellitus or controlled hypertension. Measured 84 days after the initial injection, doses of 300 mg inclisiran or more (in single or multiple doses) significantly reduced levels of PCSK9 and LDL-C. These reductions were maintained throughout 6 months. No serious adverse events were observed with inclisiran. All adverse events were classified as either mild or moderate and included mostly cough, musculoskeletal pain, nasopharyngitis, headache, back pain, and diarrhea.

## Inclisiran Lowers LDL-C Among Patients at High Cardiovascular Risk

ORION-1 was a phase 2, dose-finding trial evaluating the efficacy of different doses and dosing intervals of inclisiran among patients at high or very high CV risk with elevated LDL-C levels despite receiving the maximum dose of a statin [39••]. In this randomized, double-blind, placebo-controlled trial, a total of 501 patients were randomized to receive a single dose of placebo or 200, 300, or 500 mg of inclisiran or two doses (at day 1 and 90) of placebo or 100, 200, or 300 mg of inclisiran. Patients had to have an LDL-C level of 70 mg/dl or higher (with a history of CVD) or 100 mg/dl or higher (without a history of CVD), respectively. When entering the study, 73% of the patients were receiving a statin, and 31% of the patients were receiving ezetimibe.

Inclisiran significantly reduced LDL-C levels measured at day 180 (primary end point) in the single-dose regimens, as well as in the two-dose regimens. LDL-C levels already declined at day 14 after the first injection with a nadir at day 60 (single dose) and day 150 (two doses), respectively. The greatest reduction of LDL-C at day 180 was achieved by the two-dose 300-mg regime (least squares mean reduction: 52.6%). In this regime, 48% of the patients had an LDL-C below 50 mg/dl at day 180.

All of the patients who received two 300-mg doses of inclisiran had a decline in LDL-C at day 180 (mean absolute change of − 64.2 ± 20.7 mg/dl). In contrast, there were large variations with regard to the changes in LDL-C at day 180 among patients receiving placebo against a background statin therapy at the maximum possible dose (mean absolute change of − 0.7 ± 25.6 mg/dl).

Adverse events were reported in 76% of the patients receiving inclisiran and 76% of the patients receiving placebo. 95% of these adverse events were either mild or moderate (mostly myalgia, headache, fatigue, nasopharyngitis, back pain, hypertension, diarrhea, and dizziness) and did not differ significantly between the groups receiving inclisiran and those receiving placebo.

Transient injection site reactions were reported in 5% of the patients who received injections of inclisiran. Elevations in C-reactive protein or effects on platelet levels were not observed among patients receiving inclisiran. Transient elevations in hepatic enzyme levels were observed in three patients receiving inclisiran.

## Administering Inclisiran Every 6 Months Lowers LDL-C by Approximately 50%

Recently, two phase 3 studies, i.e., the ORION-10 and the ORION-11 trial, two randomized, double-blind, placebo-controlled, parallel-group, phase 3 trials, assessed the efficacy, safety, and adverse-event profile of inclisiran over 18 months in patients at high or very high CV risk with an LDL-C ≥ 70 mg/dl (in patient with ASCVD) or ≥ 100 mg/dl (in patients with an ASCVD risk equivalent) despite receiving a statin therapy at the maximum tolerated dose with or without ezetimibe [40••] (Table 1). ORION-10 was conducted in the USA and enrolled 1561 patients with ASCVD, whereas ORION-11 was conducted in Europe and South Africa and enrolled 1617 patients with ASCVD or an ASCVD risk equivalent (type 2 diabetes, FH, or a 10-year risk of a CV event of ≥ 20% as assessed by the Framingham Risk Score for Cardiovascular Disease or equivalent). Patients were randomized in a 1:1 ratio to receive either 284 mg of inclisiran (equivalent to 300 mg inclisiran sodium) or placebo, administered as a 1.5-ml subcutaneous injection on day 1, day 90, and every 6 months thereafter over a period of 540 days. The coprimary end points were the placebo-corrected percentage change in LDL-C from baseline to day 510 and the time-adjusted percentage change in LDL-C from baseline after day 90 up to day 540.

**Table 1:** Inclisiran: overview of phase 3 clinical trials

Trial name	Number of patients	Type of patients	Baseline LDL-C level (mg/dl)	Dosing of inclisiran sodium	Reduction in plasma LDL-C level	Follow-up	Reference
ORION-10	1.561	Patients with ASCVD	105	300 mg^1^	52%^2^	540 days	40
ORION-11	1.617	Patients with ASCVD or ASCVD risk equivalent	106	300 mg^1^	50%^2^	540 days	40
ORION-9	482	Patients with heterozygous FH	153	300 mg^1^	40%^2^	540 days	41

*ASCVD* atherosclerotic cardiovascular disease, *FH* familial hypercholesterolemia, *LDL-C* low-density lipoprotein-cholesterol

^1^Subcutaneous injection at day 1, day 90, and every 6 months thereafter

^2^At day 510

In the ORION-10 trial, patients had a baseline LDL-C of 104.7 ± 38.3 mg/dl and in the ORION-11 trial, patients had a baseline LDL-C of 105.5 ± 39.1 mg/dl. At day 510, inclisiran reduced LDL-C by 52.3% in the ORION-10 trial and by 49.9% in the ORION-11 trial, respectively. The corresponding time-adjusted percentage changes of LDL-C were 53.8% and 49.2%, respectively.

Adverse events were reported in 73.5% of the patients receiving inclisiran and in 74.8% of the patients receiving placebo in the ORION-10 trial and in 82.7% of the patients receiving inclisiran and in 81.5% of the patients receiving placebo in the ORION-11 trial. Most of the events were reported to be mild or moderate. Furthermore, laboratory results with respect to C-reactive protein and platelet count as well as the incidences of cancer-related deaths and new, worsening, or recurrent cancer were similar in the inclisiran and placebo groups in each trial. In the ORION-10 trial, serious adverse events were reported in 22.4% of the patients receiving inclisiran, including 12 deaths (1.5%), and in 26.3% of the patients receiving placebo, including 11 deaths (1.4%); in the ORION-10 trial, serious adverse events were reported in 22.3%, including 14 deaths (1.7%), of the patients receiving inclisiran and in 22.5% of the patients receiving placebo, including 15 deaths (1.9). Injection site adverse events were more frequent with inclisiran than with placebo (2.6% vs. 0.9% in the ORION-10 trial and 4.7% vs. 0.5% in the ORION-11 trial). These reactions were generally mild and non-persistent.

A prespecified exploratory end point, which included cardiac death, any signs or symptoms of cardiac arrest, non-fatal myocardial infarction, or stroke, occurred in 7.4% in the inclisiran group and in 10.2% in the placebo group in the ORION-10 trial and in 7.8% in the inclisiran group and in 10.3% in the placebo group in the ORION-11 trial.

Recently, the results of the phase 3 ORION-9 trial demonstrated the safety and efficacy of inclisiran in patients with heterozygous FH [41] (Table 1).

Furthermore, the ORION-4 trial is a dedicated ongoing CV outcome trial to examine the effect of inclisiran on cardiovascular events in patients at very high CV risk [42].

## Conclusions

Statins, in addition to non-pharmaceutical lifestyle modifications, have become the first-line therapy in preventing and treating ASCVD. Consequently, current guidelines recommend that a statin should be initiated in patients with hypercholesterolemia, but many patients, especially those at high or very high CV risk, require additional LDL-C lowering therapies [9, 10]. However, long-term adherence for statin therapy is suboptimal [43]. Inclisiran is a long-acting, synthetic siRNA; generally, RNAi-based therapies seem to require less frequent dosing than antibody therapies or other RNA-based therapies, such as antisense oligonucleotides [44].

The results of the phase 1, phase 2, and phase 3 trials with inclisiran suggest that its subcutaneous administration results in LDL-C reductions of approximately 50% [45, 46]. In the phase 3 ORION-10 and ORION-11 trials, inclisiran reduced LDL-C levels by 52.3% and 49.9% at day 510, respectively. These reductions were achieved on top of a maximum tolerated, guideline-recommended statin treatment with or without ezetimibe. When compared with PCSK9 antibody therapy, the LDL-C reduction came at a lower injection burden: while PCSK9 antibodies require one- or twice monthly injections, a regimen of subcutaneous injections on day 1, day 90, and then every 6 months was applied in the ORION-10 and the ORION-11 trials with inclisiran [40••].

In both the ORION-10 and ORION-11 trials, the prespecified CV outcome end point was reported with lower frequency in the inclisiran group when compared with the placebo group, but the total number of CV events in 540 days of trial duration was too small to yet draw definitive conclusions about effects of inclisiran on CV outcome. This question is currently being tested in an ongoing CV outcome trial—the ORION-4 trial [42].

A theoretical concern with RNA-based therapies is a potential activation of the immune system, but elevations in C-reactive protein have not yet been observed with inclisiran [39••]. Also, effects on platelet levels have not yet been observed in patients receiving inclisiran, in contrast to findings from studies with antisense oligonucleotides and other siRNA molecules [47, 48].

Injection site adverse events were more frequent with inclisiran than with placebo in all of the trials but were mild or moderate, did not require intervention, and were non-persistent, i.e., transient. Additional longer-term safety data will be provided by the results of ongoing open-label extension studies and the CV outcome trial [42].

In conclusion, subcutaneous administration of inclisiran, a synthetic siRNA directed against PCSK9, has been proven feasible. A regimen of injecting 300-mg inclisiran sodium at day 1, day 90, and every 6 months thereafter lowered LDL-C by approximately 50% in patients at high or very high CV risk or with heterozygous FH, therefore representing a promising future option for optimizing the prevention of ASCVD.
